# A distinct three-helix centipede toxin SSD609 inhibits *I*_*ks*_ channels by interacting with the KCNE1 auxiliary subunit

**DOI:** 10.1038/srep13399

**Published:** 2015-08-26

**Authors:** Peibei Sun, Fangming Wu, Ming Wen, Xingwang Yang, Chenyang Wang, Yiming Li, Shufang He, Longhua Zhang, Yun Zhang, Changlin Tian

**Affiliations:** 1 Hefei National Laboratory for Physical Sciences at the Microscale and School of Life Sciences, University of Science and Technology of China, Hefei, P. R. China; 2 High Magnetic Field Laboratory, Chinese Academy of Sciences, Hefei, P.R. China; 3 Key Laboratory of Animal Models and Human Disease Mechanisms of The Chinese Academy of Sciences & Yunnan Province, Kunming Institute of Zoology, Chinese Academy of Sciences, Kunming, Yunnan P.R. China; 4 School of Medical Engineering, Hefei University of Technology, Hefei, P. R. China; 5 Department of anesthesiology, the Second Affiliated Hospital of Anhui Medical University, Hefei, P. R. China

## Abstract

KCNE1 is a single-span transmembrane auxiliary protein that modulates the voltage-gated potassium channel KCNQ1. The KCNQ1/KCNE1 complex in cardiomyocytes exhibited slow activated potassium (*I*_*ks*_) currents. Recently, a novel 47-residue polypeptide toxin SSD609 was purified from *Scolopendra subspinipes dehaani* venom and showed *I*_*ks*_ current inhibition. Here, chemically synthesized SSD609 was shown to exert *I*_*ks*_ inhibition in extracted guinea pig cardiomyocytes and KCNQ1/KCNE1 current attenuation in CHO cells. The K^+^ current attenuation of SSD609 showed decent selectivity among different auxiliary subunits. Solution nuclear magnetic resonance analysis of SSD609 revealed a distinctive three-helix conformation that was stabilized by a new disulfide bonding pattern as well as segregated surface charge distribution. Structure-activity studies demonstrated that negatively charged Glu19 in the amphipathic extracellular helix of KCNE1 was the key residue that interacted with SSD609. The distinctive three-helix centipede toxin SSD609 is known to be the first polypeptide toxin acting on channel auxiliary subunit KCNE1, which suggests a new type of pharmacological regulation for ion channels in cardiomyocytes.

The voltage-gated potassium channels in virtually all mammalian cells regulate fluid homeostasis, nervous system signaling, and muscular contractions. These highly diverse functions are reflected in the large number of genes encoding the K^+^ channel α subunits. This multiplicity is augmented by the interaction of the K^+^ channel α subunits with a number of auxiliary subunits or β subunits^1^. Voltage-gated potassium channel auxiliary subunits play a number of roles, such as promoting α subunit surface expression early in biosynthesis, facilitating the trafficking of the protein from the endoplasmic reticulum to the cell membrane^2^ and altering aspects of the α subunit functions by modulating the channel activation, inactivation, and deactivation^3^. Some auxiliary subunits are soluble cytoplasmic proteins^4^, whereas others are transmembrane proteins, such as the MiRP (mink related peptide) family subunits^5^.

In the cardiac muscle, several potassium channels are separated into rapid delayed rectifier potassium currents (*I*_*kr*_) and slow delayed rectifier potassium current (*I*_*ks*_)^6^, which differ distinctly in their activation ranges, channel gating kinetics, rectification characteristics and pharmacological responses to various drugs^7^,^8^. KCNE1 is a single-span transmembrane auxiliary protein that modulates the voltage-gated potassium channel KCNQ1 by slowing its activation and enhancing the channel conductance to generate *I*_*ks*_ currents^9^. Changes in or modulations of the potassium channels can have both antiarrhythmic and proarrhythmic consequences^6^. Animal venoms are essentially large combinatorial libraries of bioactive molecules and hold great promise both as diagnostic tools and in the treatment of human disease^10^. Therefore, a better understanding of the interactions between venom toxins and *I*_*ks*_ channels is pharmacologically important. Venom toxins might provide excellent opportunities for developing therapies for life-threatening cardiac arrhythmias or other diseases.

Centipedes contain many diverse toxins, which are used to treat diseases in traditional Asian medicines. Recently, venomic and transcriptomic analyses of the centipede *Scolopendra subspinipes dehaani* (SSD) were conducted, and many polypeptide toxins were identified with preliminary functional annotations^11^. Among them, the novel polypeptide toxin SSD609 was purified. Primary sequencing of the purified peptide determined that SSD609 consists of 47 amino acids, and preliminary functional assays conducted with the purified peptide indicated that it targets K^+^ channels, but no detailed analysis has been conducted. Because of purity concerns and the need for a sufficient amount of SSD609 for further physiological function analysis and structural characterization using solution nuclear magnetic resonance (NMR), the toxin was chemically synthesized in this study. The successful synthesis of the peptide greatly facilitated the subsequent illustration of the detailed channel regulation functions of SSD609 and its potential target proteins. To our knowledge, SSD609 is the first peptide toxin that was determined to act on KCNE1. The results will allow for detailed structure–activity relationship studies of SSD609, which could serve as the basis for the development of potential molecular probes or drugs for probing the pathophysiological function of the KCNQ1/KCNE1 channel and related diseases, such as arrhythmia and type II diabetes.

## Results

**Figure 1:**
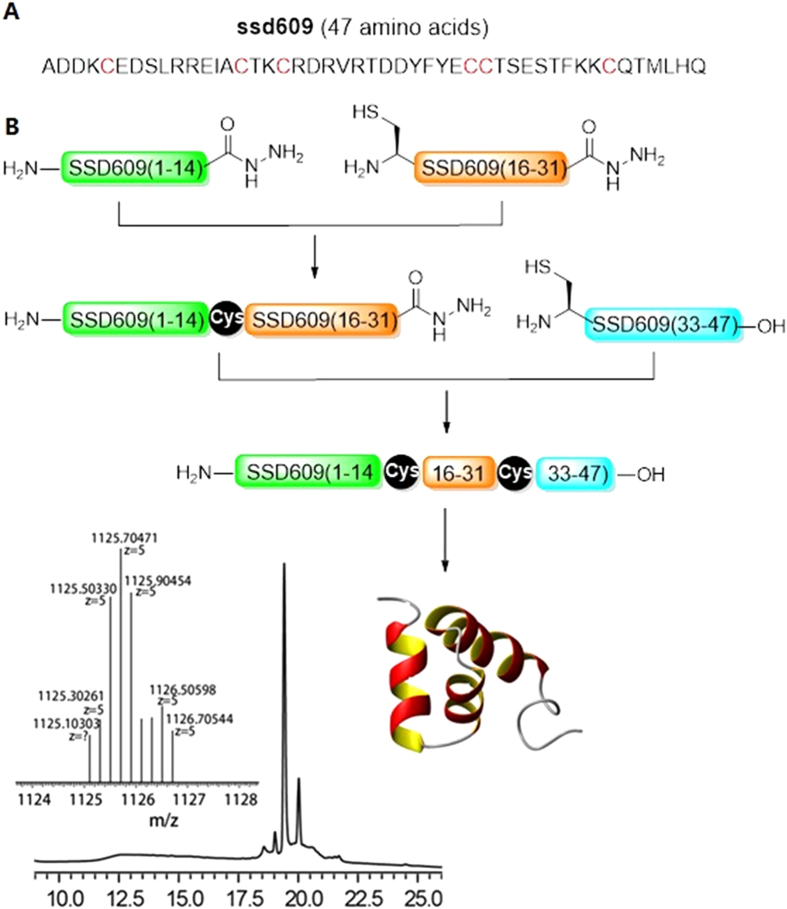
Synthesis of SSD609 using the azide switch strategy combined with hydrazide-based native chemical ligation of three peptide segments. (**A**) The primary sequence of SSD609. (**B**) Procedure for toxin ligation: (1) hydrazide-based ligation; (2) reduction with 5% Tris(2-carboxyethyl)phosphine, and (3) folding. Analytical HPLC (λ = 214 nm) and ESI–MS analyses of the final purified SSD609 were performed. Observed mass 5624.5 Da versus calculated mass 5630.3 Da (average isotopes), with six deprotonations during the formation of three disulfide bonds.

The 47-residue SSD609 toxin contains six cysteine residues, which can form three disulfide bonds (Fig. 1A). During the chemical synthesis of SSD609, one-step Fmoc solid-phase peptide chemical synthesis (SPPS) of the entire 47 residues of SSD609 was initially attempted. However, the process was unsuccessful because it was hindered by the presence of the multiple cysteine residues. Instead, the cysteine-rich toxin was synthesized using standard hydrazide-based native chemical ligation^12^, which was used to join three segments, with ligation sites at Cys15 and Cys32. Each of the three segments (SSD609(1–14)–NHNH_2_, SSD609(15–31)–NHNH_2_, and SSD609(32–47)) was synthesized with Fmoc-SPPS in the HATU-DIEA system (Fig. 1B). The three peptide segments were obtained with high yields and moderate purity. The purity and integrity of the synthesized peptides were verified by mass spectrometry (supplementary Fig. S1). The full-length SSD609 was then generated through the sequential hydrazide-based ligation of the three segments. The *in vitro* refolding of the 47-residue toxin was achieved with buffers containing oxidative/reductive reagents in a molar ratio of 1:10. The refolding process was monitored by high-performance liquid chromatography (HPLC; Supplementary Fig. S2). Ultimately, a major chromatographic fraction with three pairs of disulfide bonds was obtained and verified by mass spectrometry (insert in Fig. 1B).

**Figure 2:**
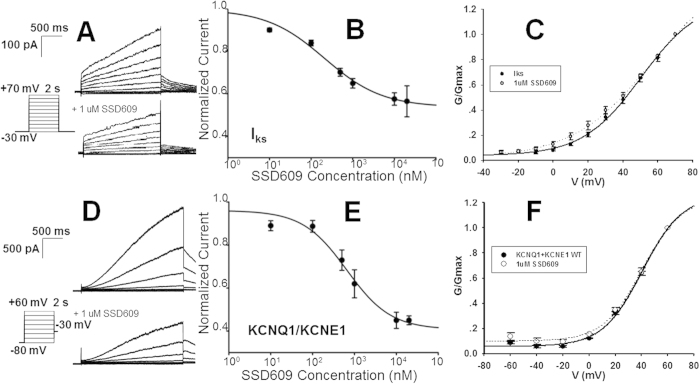
Electrophysiological characterization of KCNQ1/KCNE1 and *I*_*ks*_ in response to different voltages and toxin concentrations. (**A**) Guinea pig *I*_*ks*_ elicited by depolarizing pulses from −30 mV to +70 mV and subsequent deactivating tails at –30 mV in the absence (top) and presence (bottom) of 1 μM SSD609. (**B**) Dose–response *I*_*ks*_ channel conductance measurements at different SSD609 concentrations. IC_50_ was calculated to be 209.3 ± 60.9 nM (n = 5). (**C**) G/V curves of *I*_*ks*_ in the absence (full line, V_50_ = 50 mV, n = 7) and presence (dotted line, V_50_ = 56 mV, n = 4) of 1 μM SSD609. (**D**) KCNQ1/KCNE1 current elicited by depolarizing pulses from −80 mV to +60 mV in the absence (top) and presence (bottom) of 1 μM SSD609. (**E**) Dose–response between KCNQ1/KCNE1 channel conductance and different SSD609 concentrations. IC_50_ was calculated to be 652.7 ± 260.6 nM (n = 5). (**F**) G/V curve of KCNQ1/KCNE1 in the absence (full line, V_50_ = 40 mV, n = 5) and presence of 1 μM SSD609 (dotted line, V50 = 46 mV, n = 4).

The native toxin was unavailable in large quantities because it is normally extracted from the animal venom. Therefore, it was necessary to confirm that the chemically synthesized SSD609 was correctly folded and in the functional state. The function of the refolded SSD609 was tested using patch-clamp electrophysiology in cardiac myocytes from rats and guinea pigs. There are no *I*_*ks*_ channels in rat myocardial cells, though they are present in guinea pig myocardial cells. Therefore, the cardiac myocytes of guinea pigs were extracted to test the *I*_*ks*_ currents and the efficacy of SSD609 in inhibiting these currents (in the presence of *I*_*kr*_-inhibitory compound E4031^13^); the rat cardiac myocytes were used as the negative control (Supplementary Fig. S3). The *I*_*ks*_ tail currents were measured before the addition of SSD609 (Fig. 2A, top) in a protocol that involved depolarizing pulses from −30 to +70 mV and subsequent deactivating tails at −30 mV in the absence of SSD609. After the addition of 1 μM SSD609, approximately 50% inhibition of the *I*_*ks*_ tail current was observed (Fig. 2A, bottom). The dose–response relationship between SSD609 and the *I*_*ks*_ tail current was then tested with repetitive voltage depolarization from −30 mV to +60 mV for 2 s at 20-s intervals, with the addition of different concentrations of SSD609. A sigmoid inhibition curve was obtained and showed a concentration-dependent response. The IC_50_ of the dose-dependent SSD609 inhibition of the *I*_*ks*_ current was calculated to be 209.3 ± 60.9 nM (each value was obtained from 4–5 cells) (Fig. 2B). No obvious differences in V_50_ (the voltage at which the current is half activated) were observed in the conductance/voltage (G/V) curves in the absence or presence of 1 μM SSD609 (Fig. 2C), which indicated that SSD609 caused no dynamic changes in *I*_*ks*_ gating. In the negative control assay (patch-clamp assay of rat cardiac myocytes), no current inhibition was observed upon the addition of SSD609 (Supplementary Fig. S3). The dose-dependent *I*_*ks*_ current inhibition observed in guinea pig cardiac myocytes verified that the chemically synthesized SSD609 peptide was correctly folded and in a functional state.

Because the *I*_*ks*_ current in cardiac myocytes predominantly originates from the KCNQ1/KCNE1 channel, a patch-clamp analysis of the KCNQ1/KCNE1 channels expressed in CHO cells was performed to verify the efficacy of the chemically synthesized SSD609. As shown in Fig. 2D, approximately 50% current inhibition across KCNQ1/KCNE1 was observed when 1 μM SSD609 was added, which was similar to the inhibitory effect on the *I*_*ks*_ current. Despite its slightly lower affinity, the IC_50_ for SSD609 (652.7 ± 260.6 nM, n = 5) on the KCNQ1/KCNE1 channel (Fig. 2E) was of the same order of magnitude as the IC_50_ of SSD609 on the *I*_*ks*_ current (209.3 ± 60.9 nM, n = 5). Almost identical V_50_ values were observed in the G/V curves for the KCNQ1/KCNE1 channels in both the presence and absence of SSD609 (Fig. 2F).

**Figure 3:**
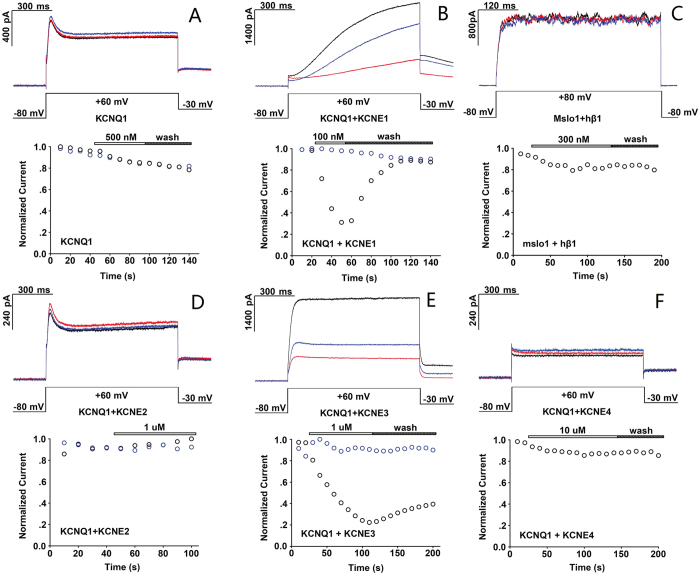
Selectivity analysis of SSD609 for different K^+^ channels. Currents before, during, and after addition of SSD609 were colored black, red, and blue, respectively (upper). The current traces were evoked by repetitive voltage depolarization from –80 mV to +60 mV (KCNQ1/KCNEs) or +80 mV (mslo1/hβ1) at 10 sec intervals (middle). The time courses for the stable currents described above are shown (bottom). The black circles indicate SSD609, while the blue circles indicate the control. (**A**) KCNQ1, (**C**) mslo1/hβ1, (**D**) KCNQ1/KCNE2 and (**F**) KCNQ1/KCNE4 channels were not inhibited by SSD609. (**B**) KCNQ1/KCNE1 and (**E**) KCNQ1/KCNE3 were reversibly inhibited by SSD609.

To further illustrate the selectivity of SSD609, Chinese hamster ovary (CHO) cells were transfected with several potassium channel systems (KCNQ1, KCNQ1/KCNE1, KCNQ1/KCNE2, KCNQ1/KCNE3, KCNQ1/KCNE4, and mslo1/hβ1). CHO cells transfected with KCNQ1 in the absence or presence of its auxiliary subunit KCNE1 were first used to assay channel inhibition by SSD609. A Y-tube perfusion system was used for rapid and local solution exchange. To monitor the time course of the channel current decline during toxin perfusion, only saline buffer (without toxin) was used as a negative control (Fig. 3, blue circle). The reversible inhibition of the KCNQ1 current by SSD609 is shown in Fig. 3A (black circles), and the inhibition of KCNQ1/KCNE1 by SSD609 is shown in Fig. 3B (black circles). Surprisingly, SSD609 had no obvious inhibitory effect on KCNQ1 alone, which indicated a possible interaction between SSD609 and the auxiliary subunit, KCNE1 (Fig. 3B).

No KCNQ1 current inhibition by SSD609 was observed (even at a higher concentration of 500 nM) (Fig. 3A), and no or only very minor inhibitory effects were exerted by SSD609 on KCNQ1/KCNE2 (Fig. 3D), KCNQ1/KCNE4 (Fig. 3F), or the BK channel plus its auxiliary subunit, mslo1/hβ1 (Fig. 3C). However, similar to the inhibitory effects of SSD609 on KCNQ1/KCNE1 (Fig. 3B), the perfusion of 1 μM SSD609 also attenuated the KCNQ1/KCNE3 currents (approximately 31.2% reversible current attenuation; Fig. 3E). However, KCNE3 normally binds to Kv3.4 and modulates the Kv3.4 currents in tissues other than cardiac myocytes, although the KCNQ1/KCNE3 pair is primarily observed in the colon^5^,^14^,^15^,^16^.

**Figure 4:**
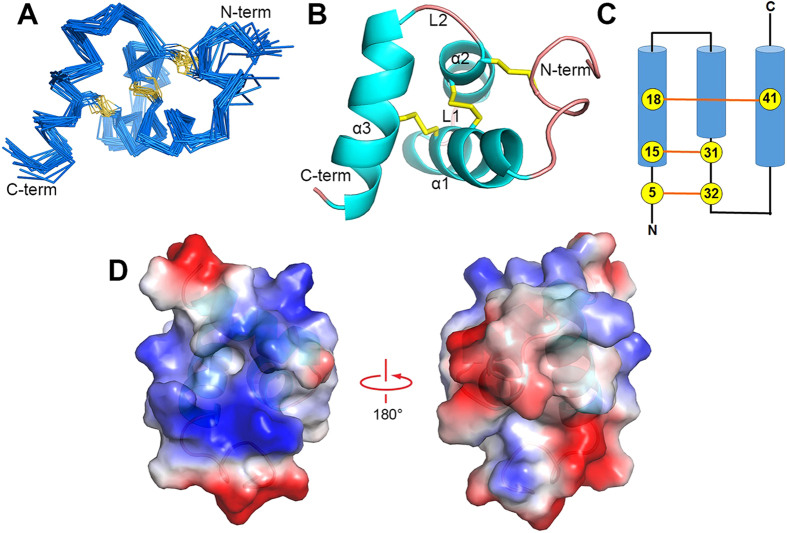
Solution NMR structure of SSD609. (**A**) Superposition of the final 20 backbone structures, with the N- and C-termini labeled. (**B**) Cartoon representation of the SSD609 structure with three disulfide bonds (yellow sticks). (**C**) Topology diagram of SSD609. Cysteine residues are shown with numbers in circles, and the disulfide bonds are indicated with orange lines. (**D**) Electrostatic potential surfaces of SSD609.

Thus, SSD609 has an explicit inhibitory effect on the *I*_*ks*_ current, which is essential for maintaining a regular heart rhythm. The structural characterization of SSD609 is essential to understanding its *I*_*ks*_-blocking mechanism. Therefore, homonuclear ^1^H–^1^H two-dimensional NMR spectra were acquired to determine the SSD609 structure in an aqueous buffer. Three sets of two-dimensional NMR spectra were obtained using a Varian 700 MHz spectrometer: double quantum filter-correlational spectroscopy (DQF-COSY), total correlation spectroscopy (TOCSY), and nuclear Overhauser effect spectroscopy (NOESY) with a mixing time of 500 ms. The resonance assignments were made using NMRPipe^17^ and analyzed using Sparky^18^. All of the backbone ^1^H resonances and over 95% of the side-chain ^1^H resonances were assigned (Supplementary Figure S5). In total, 707 inter-proton distance restraints obtained by NOESY were used to perform 200 structure calculations for SSD609 with Xplor-NIH^19^. The final 20 structures with the lowest energies were selected for structure quality assessment using PROCHECK-NMR^20^ and MOLMOL^21^ (Table S1). According to the final 20 conformational ensembles (Fig. 4A) and the representative cartoon (Fig. 4B), the toxin SSD609 consists of an irregular N-terminal helix, three α-helices (α1, Leu9–Val22; α2, Asp25–Cys32; and α3, Glu35–Leu45), and two loops between the α-helices (L1, Arg23–Thr24; and L2, Thr33–Ser34) (Fig. 4B). The three disulfide bonds that connect the six well-conserved cysteine residues of SSD609 (compared with the scolop toxin from S*colopendra mutilans* [Supplementary Figure S4]), which link the N-terminal region/α2, α1/α2, and α1/α3, stabilize the entire conformation of SSD609. The disulfide connectivity pattern is (1/5, 2/4, 3/6) in SSD609 (Fig. 4C), which differs from the normal inhibitory cysteine knot (ICK; 1/4, 2/5, 3/6)^22^ or the three-finger toxin motif^23^. The conventional ICK pattern of most toxins consists primarily of β-sheets^22^. Therefore, SSD609 might be the first reported three-helix toxin that has an unusual disulfide bond connectivity pattern, which indicates a possible new channel-modulating venom toxin.

Segregated clusters of basic (blue) or acidic residues (red) on the toxin surface can be observed in the solution NMR structure of SSD609 (Fig. 4D). As shown in Fig. 3B, SSD609 reversibly inhibited the channel conductance of *I*_*ks*_ (KCNQ1/KCNE1), specifically affecting the KCNE1 auxiliary subunit, because no inhibition of the KCNQ1 channel expressed in CHO cells was observed. The electrostatic interactions between the charged residues in the toxin and those on the channel surface have been reported to be major driving forces in toxin–channel interactions^22^. Therefore, the segregated surface charge distribution of SSD609 suggested that the positively or negatively charged surface residues of SSD609 might interact with the charged residues in the extracellular domain of KCNE1. This analysis could guide further engineering to improve the specificity or potency of the toxin as a molecular probe or drug for *I*_*ks*_-related cardiovascular diseases.

**Figure 5:**
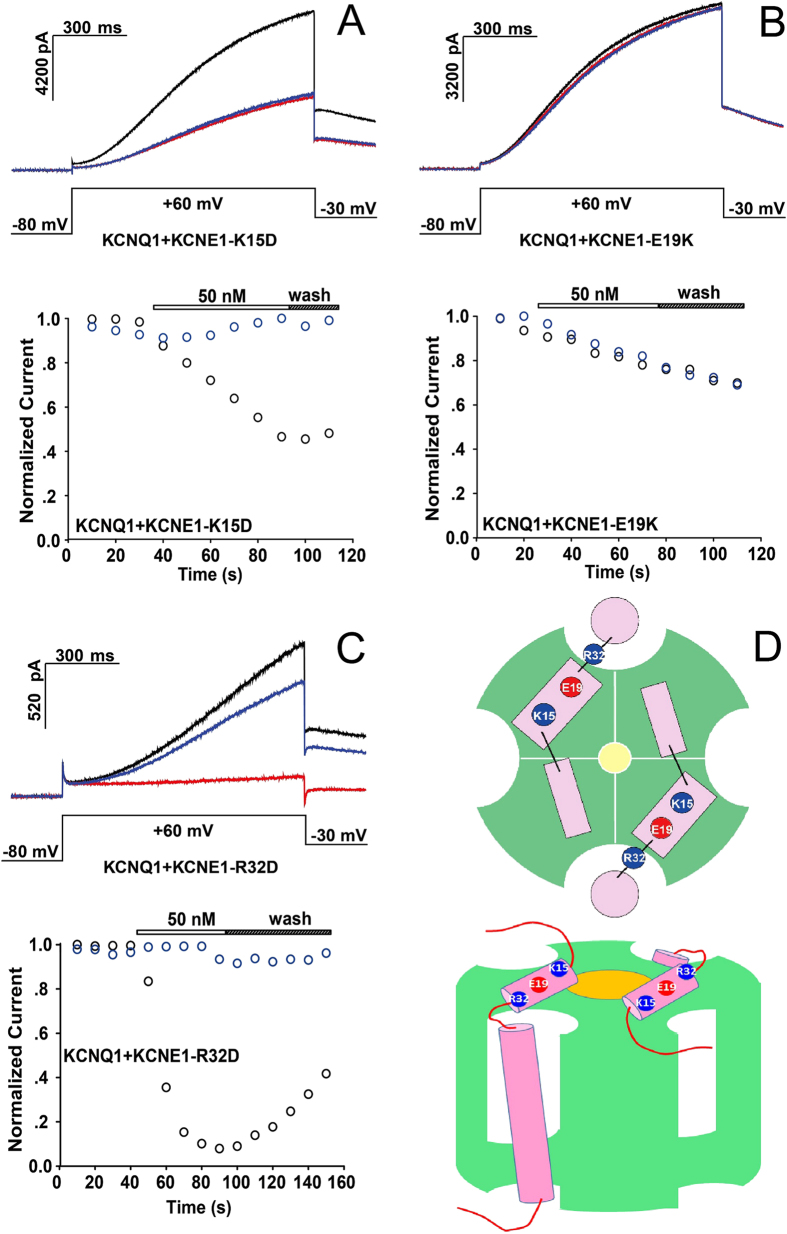
Electrophysiological analysis of SSD609 regulation for (**A**) KCNQ1/KCNE1-K15D, (**B**) KCNQ1/KCNE1-E19K, and (**C**) KCNQ1/KCNE1-R32D, in transfected CHO cells expressing the exogenous channels. Different channel currents are shown before the addition of SSD609 (black), after the addition of SSD609 (red), or after channel washing using saline buffer (blue) (upper panel). Currents were evoked with voltage steps from −80 mV to +60 mV at 10-sec intervals (middle panel). Time course of the stable current peaks is shown (bottom panel). The black circles indicate the addition of SSD609, while the blue circles indicate saline buffer alone (control). (**D**) Top view (top) and side view (bottom) of a structural schema of the KCNQ1/KCNE1 complex, from a previous report^24^. The KCNQ1 tetramer is illustrated in green. Two KCNE1 subunits are illustrated in pink. Three charged residues are shown with colored circles, representing positive (blue) or negative (red) charges.

Based on the observation of the diverse inhibitory effects of SSD609 on KCNQ1 channels with different auxiliary subunits (Fig. 3), a hypothesis can be proposed that the toxin binds to the extracellular region of the auxiliary subunit KCNE1. Recently, the solution NMR structures of KCNE1 and KCNE2 in detergent micelles demonstrated the presence of an N-terminal amphipathic helix on both, which might allow the toxin to bind to their hydrophilic sides^2^,^24^,^25^. Here, three charged residues in the N-terminal amphipathic helix of KCNE1 were mutated to residues with the opposite charges (KCNE1-K15D, KCNE1-E19K, and KCNE1-R32D), and the inhibition of their *I*_*ks*_ currents by SSD609 was analyzed. With the charge-reversing mutation KCNE1-K15D, no pronounced perturbation of the SSD609 inhibitory effect on KCNQ1/KCNE1 was observed (Fig. 5A), which indicated that residue Lys15 is not involved in the interaction between KCNE1 and the positively charged portion of SSD609, as electrostatic repulsive effects would occur. The electrophysiological results shown in Fig. 5B indicate that the mutation KCNE1-E19K abolished the inhibitory effect of SSD609, which strongly suggests that the SSD609 toxin interacts with the negatively charged residue Glu19 in the amphipathic KCNE1 N-terminal helix through the positively charged surface of the toxin (Fig. 4D). The charge-reversing mutation KCNE1-R32D did not reduce the interaction between SSD609 and KCNE1 but enhanced the inhibitory effect of the toxin (reducing the current from 30% in wild-type KCNQ1/KCNE1 to less than 10% in KCNQ1/KNCE1-R32D) (Fig. 5C), thus verifying the previously deduced interaction between the positively charged part of SSD609 and the negatively charged residues in the amphipathic N-terminal helix of KCNE1.

## Discussion

Based on a previous preliminary characterization of the KCNQ1/KCNE1 channel inhibition by centipede SSD609^11^, SSD609 was chemically synthesized using combinational SPPS and native chemical ligation methods. The chemical synthesis method not only provides abundant quantities of the toxin compared with its extraction and purification from centipedes but also specifically facilitates the formation of the disulfide bonds between the multiple pairs of cysteine residues during the production of SSD609 (Fig. 1). However, every chemically synthesized and refolded toxin should be verified as having adopted the correctly folded conformation and displaying the exact functions of the native peptide extracted from the animal venom. The inhibitory effect of the chemically synthesized SSD609 on the *I*_*ks*_ channels in cardiac myocytes of guinea pigs confirmed its correct folding and function. The specific inhibitory effect of SSD609 on the *I*_*ks*_ channel (KCNQ1/KCNE1) (Fig. 2A–C) was verified by the lack of any inhibitory effect on rat cardiac myocytes (Supplementary Fig. S3), which lack *I*_*ks*_ channels^26^, and by the direct inhibition of the exogenous KCNQ1/KCNE1 channels expressed in CHO cells (Fig. 2D–F).

To further verify the specificity of SSD609 for KCNQ1/KCNE1 and to illustrate the interactive pairing between SSD609 and the channel, the inhibitory effects of SSD609 on several potassium channel complexes containing the α subunit and different auxiliary subunits were analyzed using a reversible toxin-perfusion assay. SSD609 did not block KCNQ1 alone (Fig. 3A), KCNQ1/KCNE2 (Fig. 3D), KCNQ/KCNE4 (Fig. 3F), or the BK/β1 channel (Fig. 3C), thus confirming the specific interaction between SSD609 and KCNE1. Although SSD609 inhibited KCNQ1/KCNE3, the distribution of the KCNQ1/KCNE3 complex in the colon and other non-cardiac cells^27^ precluded any inhibitory effect of SSD609 on KCNQ1/KCNE3 in the cardiovascular system. For most toxins, the channel blockage or current inhibitory mechanism relates to the interaction between the toxin and the channel subunits^22^, and the toxin often plugs the pore region^28^,^29^. The highly specific interaction between SSD609 and the channel auxiliary subunit (KCNE1 in the *I*_*ks*_ channel) observed here is a novel and distinctive channel-current-inhibiting mechanism.

As an essential component in cardiac myocytes regulating heart rhythms, KCNQ1/KCNE1 has been the target of many chemical compounds and native polypeptides. For example, NS1643 was discovered to bind to KCNQ1 and to enhance the channel conductance^30^, whereas chromanol 293B could interact with KCNQ1 and inhibit the channel^31^. Very recently, the compound ML277 was reported to bind to the side pocket in KCNQ1, especially the KCNE1-free side pocket of KCNQ1, and to enhance the amplitude of the KCNQ1 current^32^. To date, only one polypeptide toxin, which was extracted from the scorpion, has been discovered to selectively inhibit the KCNQ1 channel (ImKTx104, K_d_ = 11.69 μM)^33^. Despite the regulatory functions of the compounds and peptide toxin, their lack of direct interaction with KCNE1 makes them not comparable with SSD609 in terms of high selectivity for the KCNQ1/KCNE1 complex. Therefore, the illustrated decent selectivity of SSD609 for KCNE1 and its related mechanism have great pharmacological significance.

The structural characterization of the centipede toxin SSD609 demonstrated that it contains a novel three-helix fold. Although animal venom peptides vary dramatically in size, from approximately less than 10 residues^34^ to more than 120 residues^35^, the most conserved structural feature of these peptides is the presence of intra-chain disulfide bonds, which stabilize the tertiary structure of the peptide. To date, more than 2,000 mature venom peptides have been reported, but only a limited number of disulfide frameworks and three-dimensional folds have been categorized. The disulfide framework of the toxin directs the three-dimensional folding and its functional interactions with heterologous receptors, voltage- or ligand-gated channels (e.g., K^+^, Na^+^, Ca^2+^, Cl^–^)^36^,^37^,^38^,^39^, transporters^40^, or enzymes^41^. Recently, six categories of consensus architectures have been proposed for venom toxin peptides. (1) The ICK motif is particularly abundant in toxins from spiders, marine cone snails, and scorpions. The ICK is composed of two or three β-sheets that are stabilized by three disulfide bonds, normally in the pattern (1/4, 2/5, 3/6)^23^,^42^. (2) The Kunitz inhibitor domain (also called the “bovine pancreatic trypsin inhibitor [BPTI] fold”) contains a conserved cysteine framework (1/6, 2/4, 3/5) and a consensus α/β fold, which are responsible for its protease inhibitor or channel-blocking activities^43^. The Kunitz domains share a similar elongated structural organization, with an α-helix in the C-terminal region connected to one of two or three central β-strands^44^,^45^. (3) The consensus core of the Kazal-like domain consists of an α-helix and a small β-hairpin that are cross-linked by disulfide bonds, with conserved disulfide connectivity (1/5, 2/4, 3/6)^46^. (4) The whey acidic protein (WAP)-type core domain contains four disulfide bonds and connects a small 3_10_ helix with the inner segment of an antiparallel β-sheet^47^. (5) The six-cysteine SXC or ShKT^48^ motif is found in very diverse organisms and is an all-α motif, with two short α-helices linked to portions of the peptide backbone *via* disulfide bonds with 1/6, 2/4, 3/5 connectivity. (6) The PLA_2_ enzymes are also abundant in animal venoms, and in this family, the enzymes normally contain more than 100 residues, with multiple cysteine residues and more complex disulfide connectivity^49^,^50^. Among the first five peptide toxin architectures, the first four categories have β-strands as major components, whereas the SXC or ShKT motif is composed of three short helices. The solution structure of SSD609 determined here contains three major α-helices and a short N-terminal helix (Fig. 4), which might place it in the SXC or ShKT motif category. However, the SXC or ShKT motif consists only of short helices, and the architecture is stabilized by (1/6, 2/4, 3/5) disulfide connectivity^48^, whereas the three major α-helices of SSD609 are stabilized by (1/5, 2/4, 3/6) disulfide connectivity (Fig. 4B,C). Therefore, the structure of centipede toxin SSD609 can be categorized as a new folding pattern, and the distinct three-helix fold of SSD609 might confer further structural specificity on its interaction with the KCNE1 auxiliary subunit.

A further patch-clamp analysis of the inhibition exerted by SSD609 on KCNQ1/KCNE1 channels with charge-reversed mutant extracellular residues in the N-terminal helix of the auxiliary subunit KCNE1 demonstrated the specific interaction between the positively charged surface of SSD609 and the negatively charged residue Glu19 of KCNE1 (Fig. 5B). Arg32 of KCNE1 might also lie in the vicinity of the interaction interface between the toxin and the auxiliary subunit (Fig. 5C). The previously reported solution NMR structure of KCNE1^24^ indicates that the amphipathic helix in its N-terminal region might be the major binding site for the toxin SSD609. However, the lack of three-dimensional structures for KCNQ1 or the KCNQ1/KCNE1 complex precludes any further docking analysis of the toxin SSD609 based on defining the exact location of the extracellular surface of KCNQ1/KCNE1. Previous docking results for KCNQ1/KCNE1 revealed a 4:2 stoichiometry for the α channel and β auxiliary subunits^24^. Thus, the KCNE1 extracellular helix might be tentatively located close to the extracellular side of KCNQ1 and in the vicinity of the channel pore. Therefore, the specific binding of SSD609 to KCNE1 (especially Glu19) might sterically hinder the diffusion of potassium ions to the pore of the KCNQ1/KCNE1 channel (Fig. 5D). Further systematic mutations introduced into SSD609 or the hypothesized interaction interface between KCNQ1 and KCNE1, with a subsequent patch-clamp-based physiological analysis, might provide clues to the exact binding sites of SSD609 and the inhibitory mechanism of SSD609 against *I*_*ks*_ channels in cardiac myocytes.

In summary, the characterization of the functions and selectivity of a chemically synthesized centipede toxin SSD609 and the determination of its structure have demonstrated its distinctive three-helix toxin architecture and its novel mechanism of potassium channel blockage, which is mediated by interaction between the toxin and the auxiliary subunit of the channel. These findings provide new insights and a basis for the in-depth illustration of KCNQ1/KCNE1 channel functions and their pathophysiological relevance, as well as a potential new type of molecular template for drug development to treat related human diseases, such as cardiovascular diseases and diabetes.

## Methods

### Chemicals

All chemical reagents were obtained from commercial sources Sigma-Aldrich (Missouri, USA), Energy-Chemical (Shanghai, China), J&K (Shanghai, China) and TCI (Tokyo, Japan) and used as received. Organic solvents were obtained from Sinopharm Chemical Reagent Co., Ltd. (Shanghai, China)

### SSD609 chemical synthesis, native chemical ligation

As shown in Fig. 1, we used native chemical ligation to connect the three segments of SSD609 in sequence. The three segments were synthesized according to the protocol given in the Supplementary Material. The first (amino acids 1–14) and second (amino acids 15–31) segments were joined first. The hydrazide was reacted with NaNO_2_ to produce azide, which generated a thioester after the addition of 4-mercaptophenylacetic acid, which was then processed as described for common native chemical ligation. The ligation product was purified by HPLC, lyophilized, and prepared for the next ligation reaction. The second ligation was identical to the first, and after ligation, the final product was purified and lyophilized.

### SSD609 refolding

SSD609 was lyophilized after purification with HPLC. A weakly alkaline solution (0.33 M ammonium acetate [NH_4_OAc], 0.5 M guanidine hydrochloride [GnHCl], and reduced glutathione [GSH]: oxidized glutathione [GSSG]: peptide = 100:10:1, pH 7.8) was added to a final SSD609 concentration of 100 μM and stored at 4 °C for 24 h. GnHCl was used as a denaturant to prevent protein aggregation. GSH and GSSG can simulate physiological conditions, inducing nonnative disulfide bonds to fold into a thermodynamically stable conformation. The liquid mixture was purified again by HPLC to produce a uniform conformation. The peptide was lyophilized for subsequent experiments.

### Plasmid construction

The coding sequences for wild-type human KCNQ1, KCNE1, KCNE2, KCNE3, KCNE4, and mslo1/hβ1 were subcloned into the pcDNA3.1/Zeo(+) vector. All site-directed mutations (KCNE1-K15D, KCNE1-E19K, and KCNE1-R32D) were generated by overlap PCR using PrimeSTAR HS (TaKaRa, Japan), and inserted into pcDNA3.1/Zeo(+). All primers were produced by Sangon. The mutants were sequenced to verify that no unwanted mutations had been introduced.

### Cell culture and transient transfection of CHO cells

Chinese hamster ovary (CHO) cells were cultured in DMEM/F12 medium (Gibco) supplemented with 10% fetal bovine serum (FBS), 100 U/mL penicillin, and 100 U/mL streptomycin at 37 °C in a 5% CO_2_ incubator. The CHO cells were then transferred to 24-well plates for transfection. Upon reaching 90% confluence, the CHO cells were transfected with 0.6 μg of plasmid encoding EGFP, 0.8 μg of plasmid encoding KCNQ1 or mslo1, and 0.8 μg of plasmids encoding the auxiliary subunits using Lipofectamine 2000 reagent (Invitrogen, USA). After incubation for 4–5 h, the cells were transferred to poly-l-lysine (Sigma)-coated slides and cultured for another 24 h in fresh medium. They were then used for the electrophysiological experiments.

### Electrophysiological analysis of CHO cells

For the whole-cell recordings, the bath solution contained (in mM): 150 NaCl, 4 KCl, 2 CaCl_2_, 1 MgCl_2_, and 10 HEPES (pH 7.4, 310 mOsm). The electrodes were pulled from thick-walled borosilicate glass capillaries with filaments (1.5 mm diameter; Sutter Instruments) on a four-stage puller (P-1000; Sutter, USA) and had resistances of 2–5 MΩ when they were filled with intracellular solution containing (in mM): 140 KCl, 10 NaCl, 5 EGTA, 10 HEPES, 1 MgATP, and 0.2 NaGTP (pH 7.4, 295 mOsm). MgATP and NaGTP were used to prevent KCNQ1 current rundown. All chemicals were obtained from Sigma. The experiments were performed at room temperature using an EPC-10 amplifier (HEKA Electronic) with the data acquisition software PatchMaster. The cell membrane potential was maintained at −80 mV, and the currents were elicited by a trace of depolarizing pulses of identical voltage once every 10 s. SSD609 was added when the currents were stable. Cells that showed a change in resistance or capacitance of more than 10% during the experiment were discarded. The data were analyzed using Clampfit and SigmaPlot.

### Isolation of guinea pig cardiac myocytes

Male guinea pigs (300–400 g) were heparinized (10 units/g) for 15 min before being injected with sodium pentobarbital (60 mg/kg) to induce anesthesia. Their hearts were excised and perfused rapidly with calcium-free perfusion buffer. The Langendorff system, which was maintained at 37 °C, was used in the perfusion process. After 5 min, the blood was washed from the hearts, and collagenase type II (1 mg/mL in perfusion buffer) was perfused for approximately 20 min to digest the cardiac myocytes. The ventricles were cut off and digestion stopped using 10% FBS in perfusion buffer. After the cardiac myocytes were mechanically dispersed, calcium was reintroduced to a final concentration of 1 mM. The cells were then used for the patch-clamp experiment. The calcium-free perfusion buffer contained (in mM): 120.4 NaCl, 14.7 KCl, 0.6 KH_2_PO_4_, 0.6 Na_2_HPO_4_, 1.2 MgSO_4_, 10 HEPES, 4.6 NaHCO_3_, 30 taurine, 10 2,3-butanedione monoxine, and 5.5 glucose.

### Cardiac myocyte patch-clamp experiment

For whole-cell recordings of the guinea pig cardiac myocytes, the bath solution was as described above, except that it also contained 0.1 mM CdCl_2_ to block L-type I_Ca_, 1 mM BaCl_2_ to block I_KI_, and 5 μM E-4031 to block I_Kr_. The pipette solution contained (in mM): 125 potassium aspartate, 20 KCl, 1 MgCl_2_, 1 MgATP, 0.5 NaGTP, 10 EGTA, and 5 HEPES. The holding potential was maintained at −30 mV to inactivate I_Na_ and T-type I_Ca_, and the currents were elicited by voltage steps from −30 mV to +70 mV in 10 mV increments for 2000 ms, followed by a return to −30 mV. The step current contained other potassium currents, whereas the tail current was I_ks_. To test the efficacy of SSD609, the current traces were evoked by repetitive voltage depolarization from −30 mV to +60 mV for 2 s at 20 s intervals, and the tail currents were analyzed. SSD609 was added after the currents were stable. Cells that showed a change in resistance or capacitance of more than 10% during the experiment were discarded.

### Patch-clamp electrophysiological data analysis

The data were analyzed using Clampfit and SigmaPlot. The dose–response curves used to determine the IC_50_ values were fitted using the Hill equation: y = 1 + (I_max_ − 1)/(1 + (IC_50_/x)^h^), where x is the toxin concentration, h is the Hill coefficient, and IC_50_ is the half-maximal effect. The results are presented as the mean ± standard error (SE), and n is the number of experiments.

### Solution NMR structure determination of SSD609

The resonances were assigned based on three homonuclear spectra, DQF-COSY, TOCSY, and NOESY (mixing time, 500 ms), which were collected on a Varian 700 MHz spectrometer. The NMR spectra were processed using NMRPipe^17^ and analyzed using Sparky^18^. The distance restraints obtained by NOESY were applied to the calculation of the SSD609 structure using Xplor-NIH^19^. Two hundred structures were calculated, and the 20 structures with the lowest energies were selected to represent the structure of SSD609. The quality of the structures was assessed using PROCHECK-NMR^20^ and MOLMOL^21^.

## Additional Information

**How to cite this article**: Sun, P. *et al.* A distinct three-helix centipede toxin SSD609 inhibits *I*_*ks*_ channels by interacting with the KCNE1 auxiliary subunit. *Sci. Rep.* **5**, 13399; doi: 10.1038/srep13399 (2015).

## Supplementary Material

Supplementary Information
