# Supplementary material for: A distinct three-helix centipede toxin SSD609 inhibits Iks channels by interacting with the KCNE1 auxiliary subunit
Source: Sci Rep. 2015 Aug 26;5:13399. doi: 10.1038/srep13399 (PMC4549624; doi:10.1038/srep13399)
Supplement: Supplementary Information [file srep13399-s1.doc]

A distinct three-helix centipede toxin SSD609 inhibits *I_ks_* channels by interacting with the KCNE1 auxiliary subunit

Peibei Sun^1,#^, Fangming Wu^2,#^, Ming Wen^1^, Xingwang Yang^3^, Chenyang Wang^1^, Yiming Li^4^, ShuFang He^5^, Longhua Zhang^1^,*, Yun Zhang^3^,* and Changlin Tian^1,2^,*

1. Hefei National Laboratory for Physical Sciences at the Microscale and School of Life Sciences, University of Science and Technology of China, Hefei, P. R. China

2. High Magnetic Field Laboratory, Chinese Academy of Sciences, Hefei, P.R. China

3. Key Laboratory of Animal Models and Human Disease Mechanisms of The Chinese Academy of Sciences & Yunnan Province, Kunming Institute of Zoology, Chinese Academy of Sciences, Kunming, Yunnan P.R. China

4. School of Medical Engineering, Hefei University of Technology, Hefei, P. R. China

5. Department of anesthesiology, the Second Affiliated Hospital of Anhui Medical University, Hefei, P. R. China

^#^ These authors contributed equally in this work.

* Correspondence and requests for materials should be addressed to L.Z., Y.Z. or C.T. (Email: [zlhustc@ustc.edu.cn](mailto:zlhustc@ustc.edu.cn), [zhangy@mail.kiz.ac.cn](mailto:zhangy@mail.kiz.ac.cn) or [cltian@ustc.edu.cn](mailto:cltian@ustc.edu.cn))

**Peptide synthesis protocol:**

The SSD609 was synthesized using combinational SPPS and native chemical ligations between synthesized peptide fragments. Specific steps are shown below:

1. Weight 2-Cl-Trt resin 500 mg (substitution 0.56 mg/mmol) to the reaction vessel (RV).

2. Swell the resin with mixed DMF and DCM (1:1) in 20min.

3. Drain the liquid over, add AA (4eq) + DCM + DIEA (8eq) to the RV, and oscillate 1h for the First peptide ligation. After the ligation, Drain over, wash 5 time (DMF), use 5% CH_3_OH to seal the unreacted 2-Cl-Trt resin.

4. Use 20% piperidine (DMF, 0.1Moxymainside) for deprotection, 2min+10min.

5. Wash the resin with DMF(5×) + DCM(5×) + DMF(5×)，then come another ligation HATU(4eq) + DIEA(8eq) system.

6. Do the same above until the 10th AA, and then twice coupling is used.

7. After the last deprotection, Wash with DMF(5×)+DCM(5×), swab-off, add the cleavage reagents(Reagent K), oscillate 3h, nitrogen blow and Ether precipitate, crude product is got.

8. Crude product is Purified by HPLC, freezed with liquid N2, then lyophilized, and eventually product received.

Note: if the C-terminal is hydrazide, pick the Fmoc-NH-NH2-trt resin (prepared in advance) instead of 2-Cl-Trt resin, swell and do deprotection as above, also the coupling and other steps.

**Figure S1**: MS spectra verification of the synthesized SSD609 fragments.

ssd609(1-14)-NHNH_2_


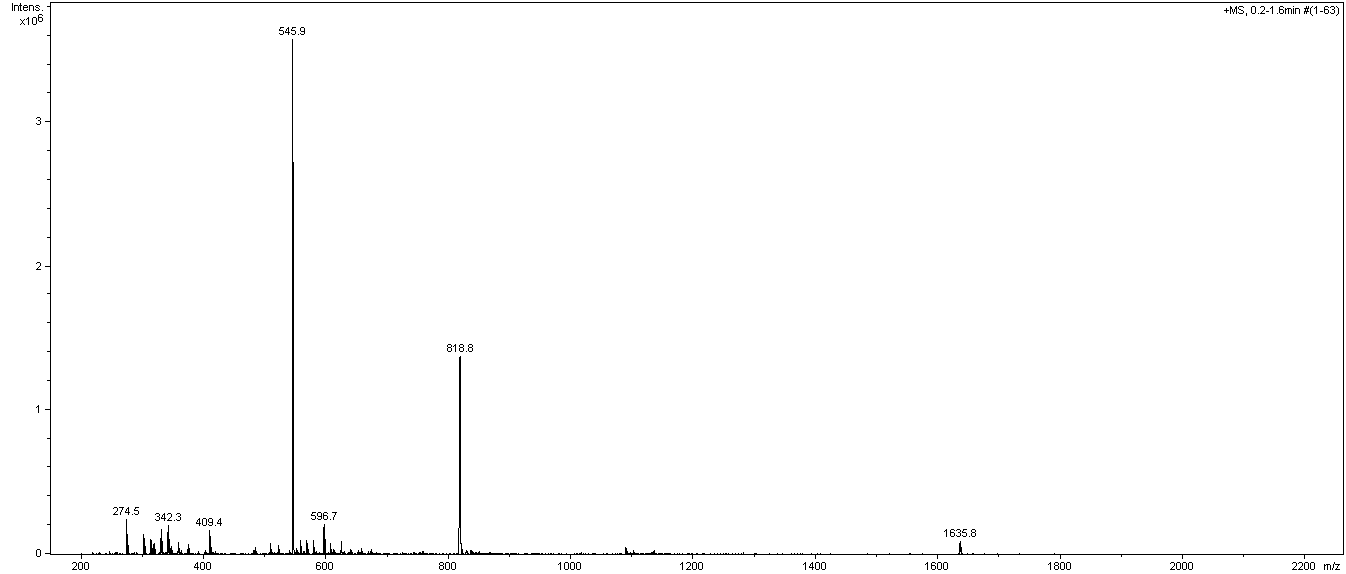


ssd609(15-31)-NHNH2


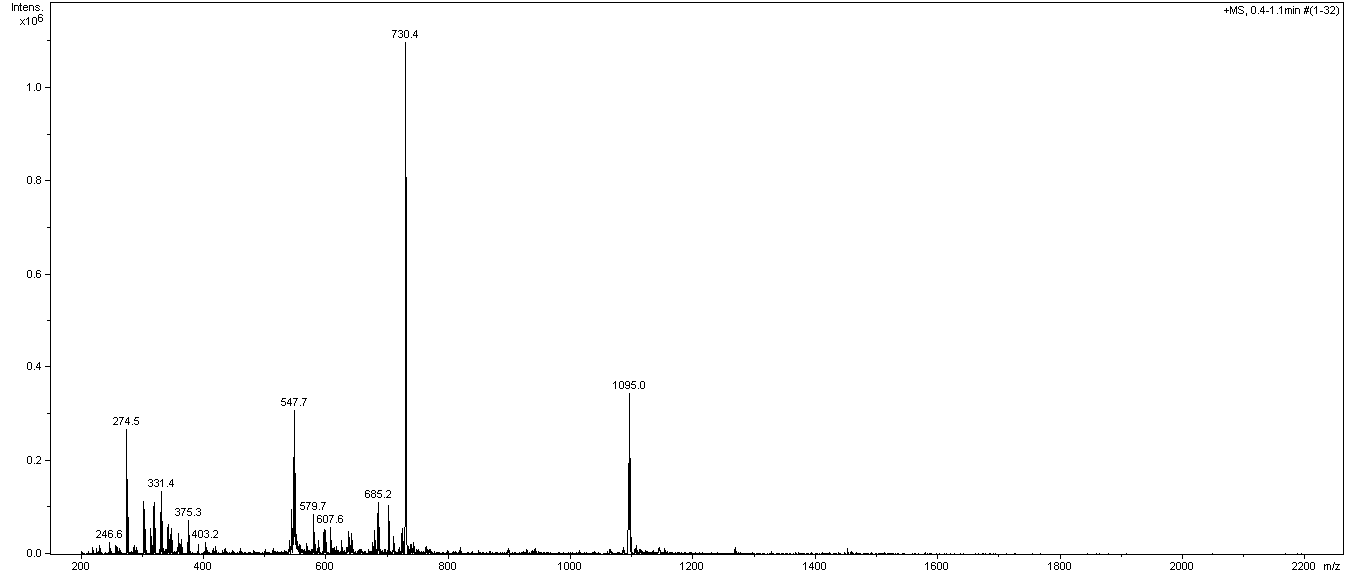


ssd609(32-47)


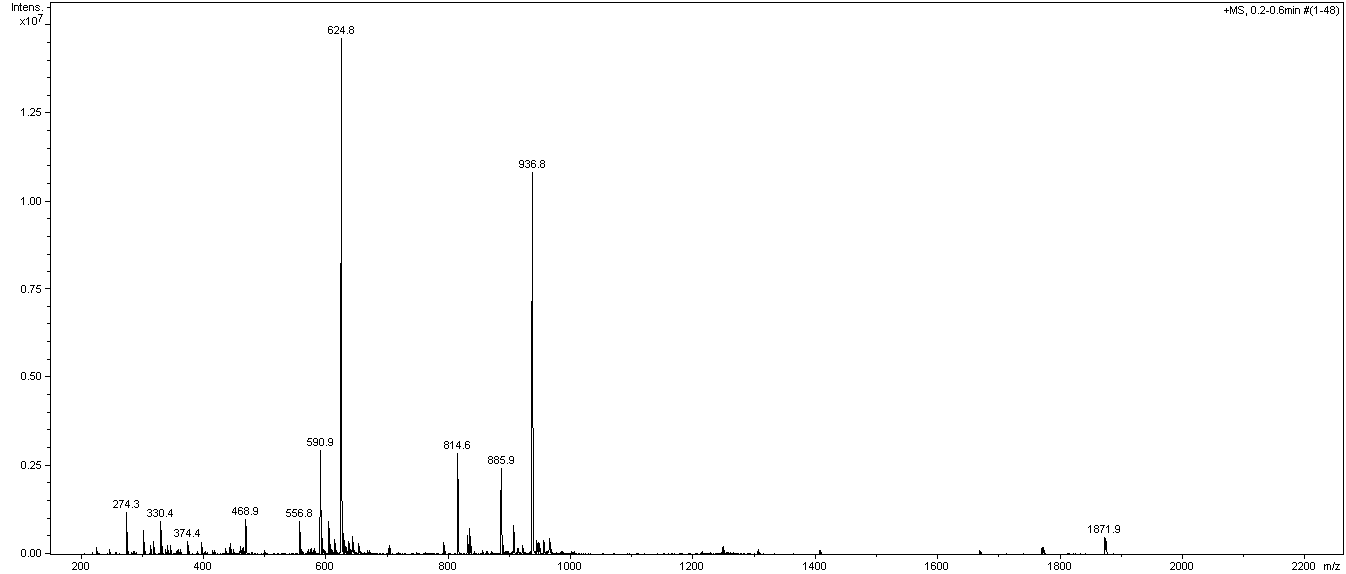


**Figure S2**: Folding of SSD609. HPLC chromatograms was applied to monitor protein refolding. (Left) before and (Right) after refolding of SSD609.


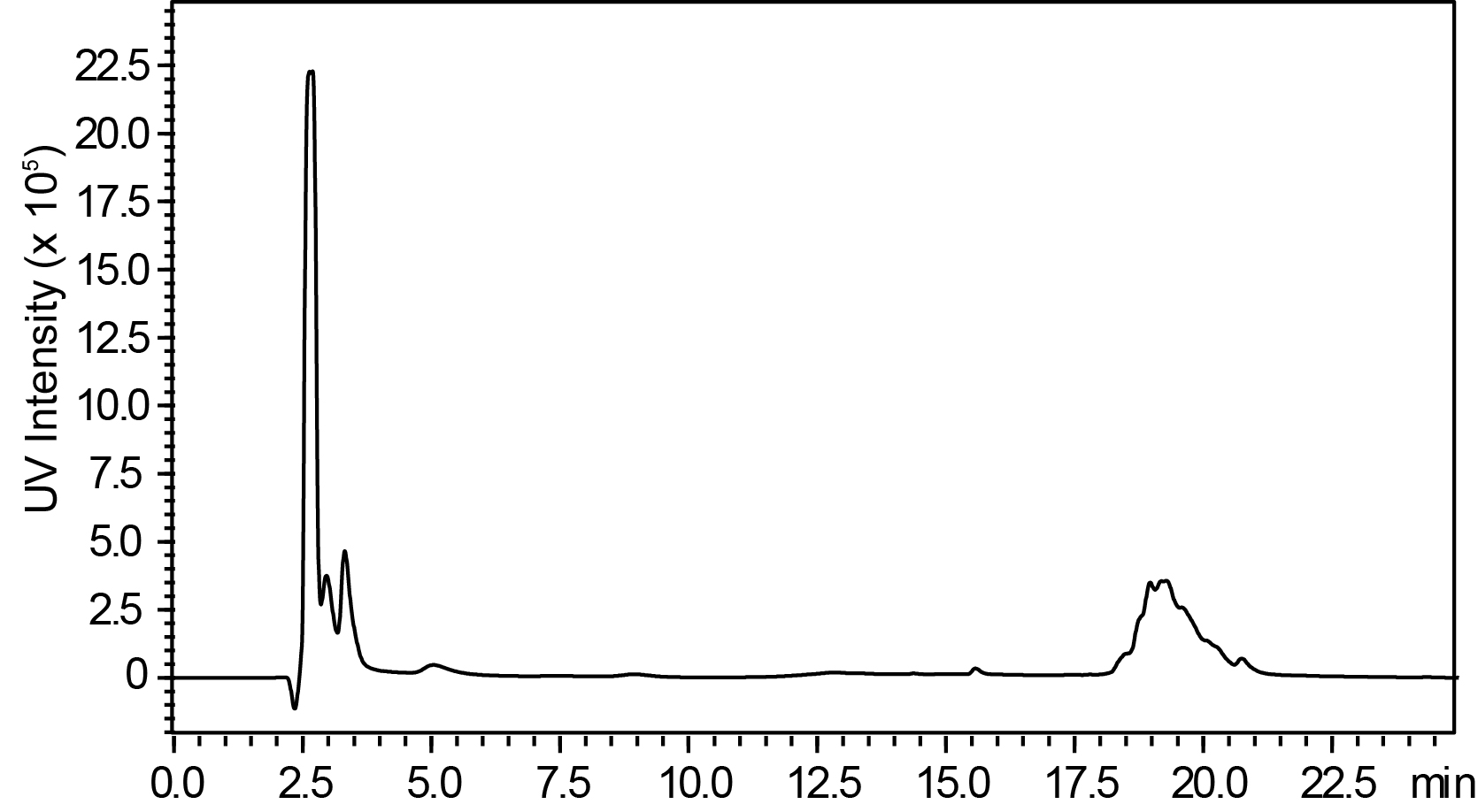

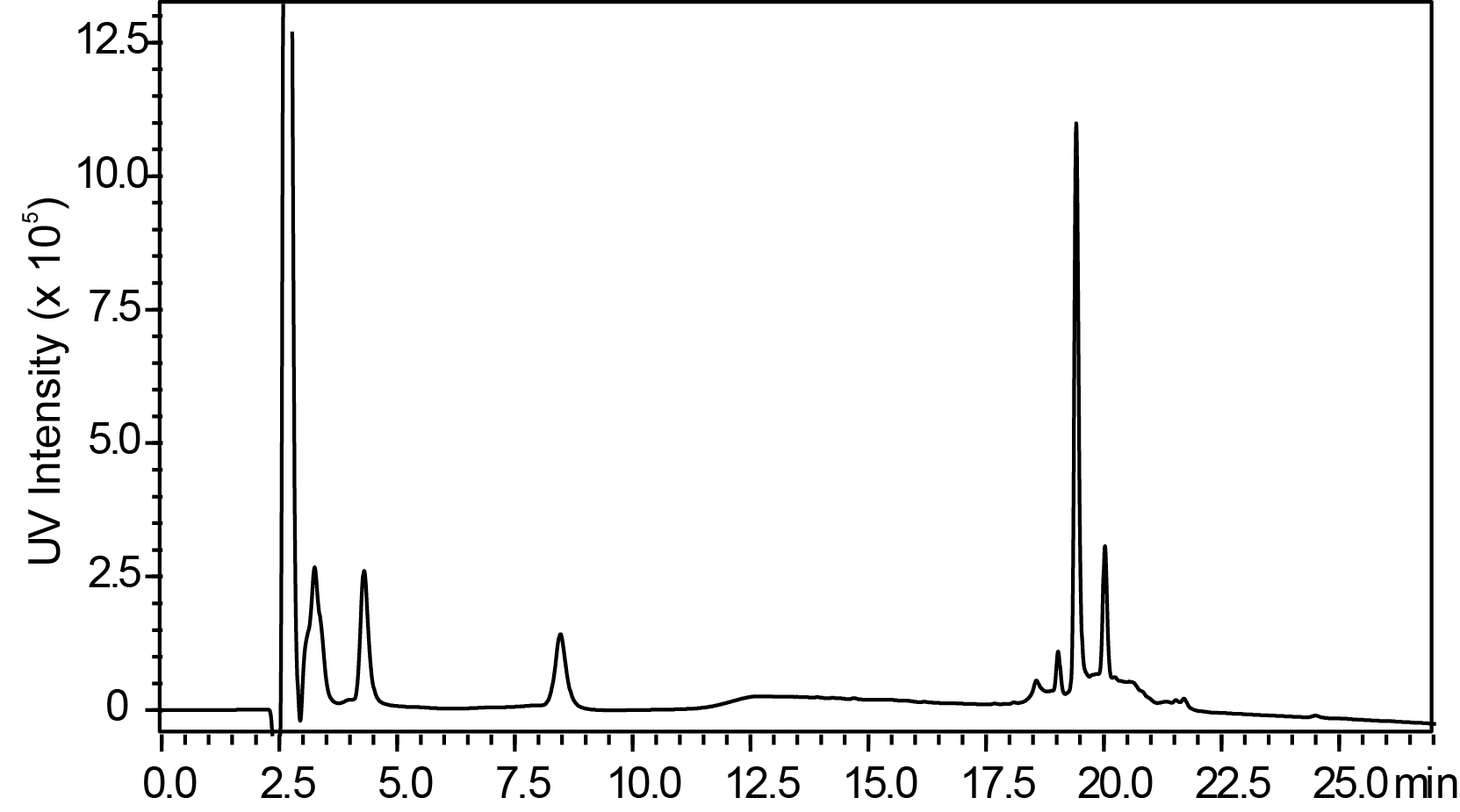


**Figure S3**: SSD609 has no effects on Rat myocardial K^+^ current. (A) Rat myocardial K+ current elicited by depolarizing pulses from -80 to +60 mV. (B) The traced line coloured in black and red represented acquired K^+^ channel currents before and during 1μM SSD609 application. (C)Time course of stable current peaks. Black circles stand for addition of SSD609.


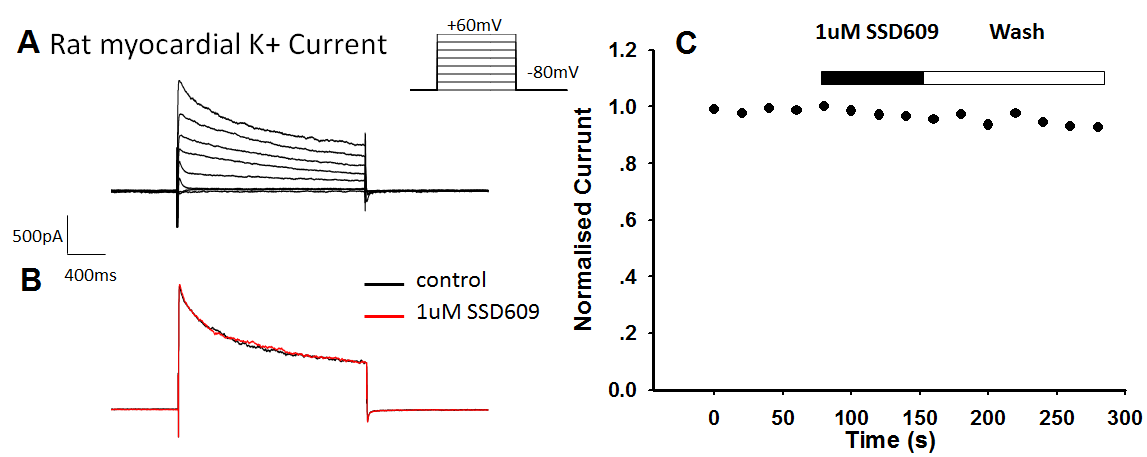


**Figure S4:** Multiple sequence alignment of several scoloptoxins from Scolopendra mutilans. The secondary structures of SSD609 are labeled above the sequences. The conserved cysteins and the disulfide-bonds are indicated by green numbers.


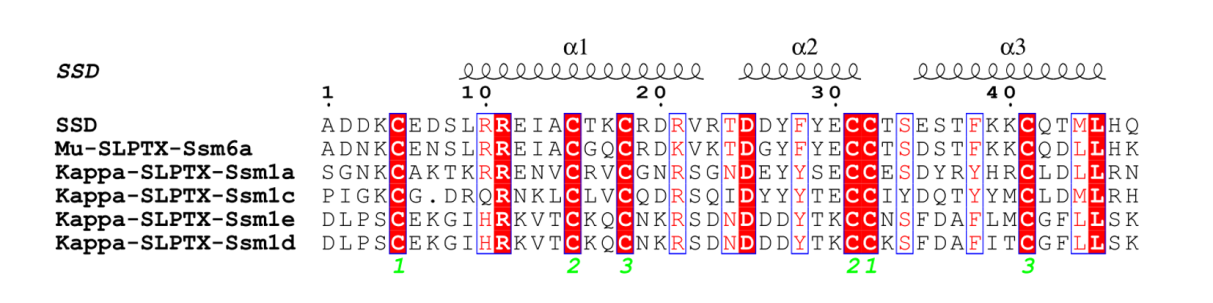


**Figure S5:** ^1^H-^1^H NOESY spectrum with 500 ms mixing time. The backbone and sidechain 1H resonance assignment were achieved through analyzing the DQF-COSY, TOCSY and NOESY spectra.


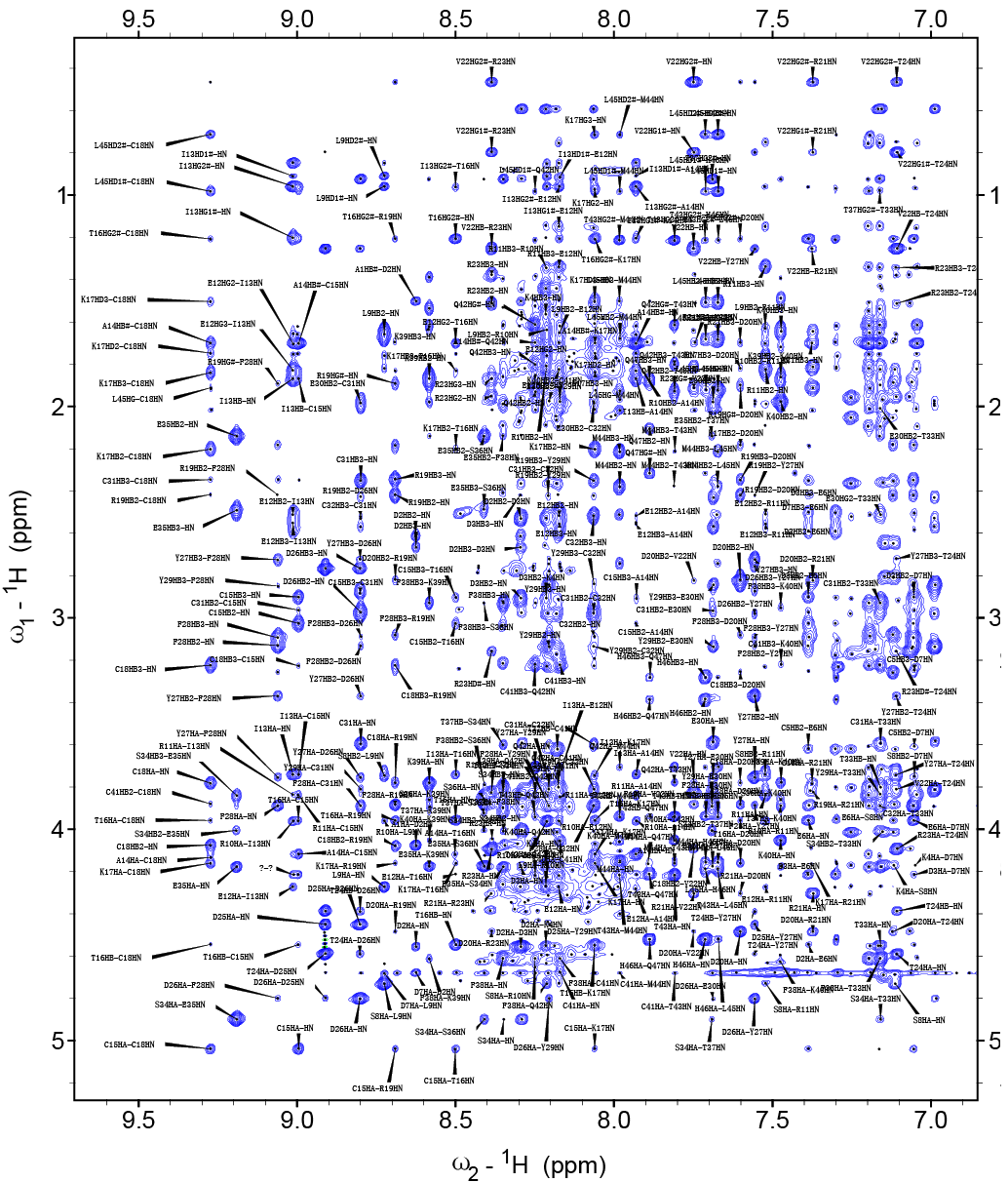


**Table S1.** Structural statistics for the final 20 conformers of SSD609.

| **Number of Distance Constrains** |  |
| --- | --- |
| NOE distance constraints | 715 |
| intraresidue ( *i* – *j* = 0) | 158 |
| sequential ( *i* – *j* = 1) | 248 |
| medium-range ( 2≤ *i* – *j* ≤ 4) | 260 |
| long-range ( *i* – *j* ≥ 5) | 49 |
| **Rmsd for experimental restraints** |  |
| NOE distance constraints | 0.0051±0.0012 |
| Dihedral Angle Constraints | 0.0000±0.0000 |
| **Rmsd from idealized covalent geometry** |  |
| Bonds (Å) | 0.0007±0.0001 |
| Angles (°) | 0.3434±0.0028 |
| Impropers (°) | 0.1788±0.0046 |
| **Average RMSD of Atomic Coordinates (Å)** |  |
| backbone atoms (secondary structure region^a^) | 0.610 |
| all heavy atoms (secondary structure region^a^) | 1.281 |
| **Ramachandran plot analysis (%)** |  |
| most favorable region | 80.3 |
| additional allowed regions | 15.0 |
| generously allowed regions | 4.4 |
| disallowed regions | 0.2 |

^a^ Secondary structure region: residues 9-22, 25-32, and 35-45.
